# Combined effects of host genetics and diet on porcine intestinal fungi and their pathogenic genes

**DOI:** 10.3389/fmicb.2023.1192288

**Published:** 2023-09-25

**Authors:** Tao Wang, Jiahao Liu, Yuheng Luo, Bing Yu, Xiangfeng Kong, Ping Zheng, Zhiqing Huang, Xiangbing Mao, Jie Yu, Junqiu Luo, Hui Yan, Jun He

**Affiliations:** ^1^Institute of Animal Nutrition, Sichuan Agricultural University, Chengdu, China; ^2^Key Laboratory of Animal Disease-resistant Nutrition, Chengdu, China; ^3^Institute of Subtropical Agriculture, Chinese Academy of Sciences, Changsha, China

**Keywords:** genetics, fungi, metagenomic, diet fiber, pigs

## Abstract

As research on gut microbes progresses, it becomes increasingly clear that a small family of microbiota--fungi, plays a crucial role in animal health. However, little is known about the fungal composition in the pig intestine, especially after a dietary fiber diet and hybrid genetics, and the changes in host pathogenicity-associated genes they carry. The purpose of this study is to investigate the effects of diet and genetics on the diversity and structure of porcine intestinal fungi and to describe, for the first time, the host pathogenicity-related genes carried by porcine intestinal fungi. Samples of colonic contents were collected for metagenomic analysis using a 3 × 2 parsing design, where three pig breeds (Taoyuan, Duroc, and crossbred Xiangcun) were fed high or low fiber diets (*n* = 10). In all samples, we identified a total of 281 identifiable fungal genera, with *Ascomycota* and *Microsporidia* being the most abundant fungi. Compared to Duroc pigs, Taoyuan and Xiangcun pigs had higher fungal richness. Interestingly, the fiber diet significantly reduced the abundance of the pathogenic fungus *Mucor* and significantly increased the abundance of the fiber digestion-associated fungus *Neocallimastix*. Pathogenic fungi exert their pathogenicity through the genes they carry that are associated with host pathogenicity. Therefore, we obtained 839 pathogenicity genes carried by the spectrum of fungi in the pig intestine by comparing the PHI-base database. Our results showed that fungi in the colon of Taoyuan pigs carried the highest abundance of different classes of host pathogenicity-related genes, and the lowest in Duroc pigs. Specifically, Taoyuan pigs carried high abundance of animal pathogenicity-related genes (*CaTUP1*, *CPAR2_106400*, *CaCDC35*, *Tfp1*, *CaMNT2*), and *CaTUP1* was the key gene for Candida pathogenicity. The intestinal fungal composition of crossbred Xiangcun pigs and the abundance of host pathogenicity-associated genes they carried exhibited a mixture of characteristics of Taoyuan and Duroc pigs. In conclusion, our results provide the first comprehensive report on the effects of dietary fiber and genetics on the composition of intestinal fungi and the host-associated pathogenicity genes they carry in pigs. These findings provide a reference for subsequent pig breeding and development of anti-pathogenic fungal drugs.

## Introduction

In recent years, the role of microorganisms in the animal gut has received increasing attention as research has been intensively explored. However, the role of fungi, which account for only 0.1% of gut microbes, has not received much attention from researchers (Qin et al., 2010), and the lack of research is partly due to the low relative abundance of fungi and the lack of a genome with a clear reference (Sokol et al., 2017). Nevertheless, with the development of high-throughput sequencing technologies, methodological difficulties associated with the study of intestinal fungi have been alleviated in recent years. A growing body of research has confirmed that fungi play a critical role in various physiological processes of the host, including immunity, digestion, and metabolism (Luo et al., 2021; Wang et al., 2023). Diseases such as colon cancer, IBD and cirrhosis have been shown to cause dysbiosis of the intestinal fungal flora, such as an increase in the *Basidiomycete*/*Ascomycete* ratio and the abundance of *Candida albicans* (Botschuijver et al., 2017; Sokol et al., 2017; Bajaj et al., 2018; Coker et al., 2019). Interestingly, commensal fungi have been shown to reduce susceptibility to diseases caused by bacterial depletion and have the potential to functionally replace intestinal bacteria to maintain host immune responses in the gut (Jiang et al., 2017). These findings suggest that fungi, even though they constitute only “0.1%” of the gut microbial composition, play a significant role in host immunity, intestinal health, and homeostasis. It has been demonstrated that approximately 500 fungal species in nature are pathogenic to humans (Köhler et al., 2017). The pathogenicity of fungi is directly related to the genes they carry that are associated with host pathogenicity, and these genes are also crucial for immune escape and pathogenic invasion of pathogenic fungi (Moran et al., 2012; Köhler et al., 2017; Siscar-Lewin et al., 2022). For example, *Candida albicans* invades host tissues through its secreted peptide toxin, which crosses the epithelial barrier, causing cellular damage through Candida lysins (Moyes et al., 2016; Allert et al., 2018; Naglik et al., 2019).

Pigs exhibit numerous similarities with humans in terms of anatomical size and structure, immunology, genome, and physiology. These resemblances render them invaluable animal models for various clinical research applications (Meurens et al., 2012). Therefore, a comprehensive dissection of the fungal composition and diversity of the porcine intestine is crucial to enhance the potential of this species model. Previous studies have revealed *Ascomycota* and *Basidiomycota* as the two predominant fungal phyla in the porcine intestine (Hu et al., 2016), which is consistent with earlier findings in the human intestine. Fungal abundance and species composition in the gut are influenced by host genetics, diet, and bacterial microbiota (Lai et al., 2019). For instance, the digestion of different structural starches has been linked to the abundance of low abundance fungi in the pig intestine, such as *Eurotium* and *Microascus* (Luo et al., 2021). Furthermore, significant variations have been observed in the fungal composition in the intestine of adult, Yorkshire, and Tibetan pigs with different genetic backgrounds (Li et al., 2020). However, there is currently no evidence regarding the effects of fiber diet and crossbreeding genetics on changes in the fungal communities in the intestinal tract of pigs. To address this gap, we selected local Chinese pig breeds, including Taoyuan pigs, Duroc pigs, and Xiangcun pigs, a crossbreed of the two, as models to explore changes in the structure of their intestinal fungal communities through a high and low fiber diet, followed by the collection of their colonic contents.

The Taoyuan pig is a unique local breed in China, primarily distributed in Taoyuan County, Hunan Province. Xiangcun pigs, a crossbreed of Taoyuan and Duroc pigs, inherit Duroc’s high reproductive performance and acquire Taoyuan pigs’ high-quality meat, roughage tolerance, and disease resistance (Ding et al., 2022; Liu et al., 2022). However, the fungal composition and distribution of host pathogenicity-related genes carried in the intestine of Taoyuan and crossbred Xiangcun pigs remain unreported. In this study, we conducted a macrogenomic analysis to compare the differences in fungal composition and distribution in the colon of different pig breeds (Taoyuan, Xiangcun, and Duroc) under high and low fiber diets. Additionally, we comprehensively analyzed the spectrum of host pathogenicity-related genes carried by porcine intestinal fungi. Our study provides a comprehensive exploration of the effects of diet and genetics on the distribution of intestinal fungi in pigs and investigates the differences in pathogenic genes carried by intestinal fungi.

## Materials and methods

The management of animal experiments involved in the research shall refer to the “Regulations on the Administration of Laboratory Animals” (Ministry of Science and Technology, China, revised in June 2004). Sample collection was approved by the Institutional Animal Care and Use Committee of Sichuan Agricultural University, Sichuan, China (No. 20181105).

### Animal trial and sample collection

This experiment adopted a 3 × 2 factorial design, 3 breeds of pigs (*n* = 10; Age: 60 days; Taoyuan pig: 13.87 ± 0.58 kg, Purchased from Xiangcun High-tech Agriculture Co., Ltd.; Duroc pig: 18.50 ± 1.09 kg, Purchased from Linli Tianxin Seed Industry Co., Ltd.; Xiangcun pigs: 14.47 ± 0.15 kg; Purchased from Xiangcun High-tech Agriculture Co., Ltd.) were fed with high-fiber feeds (Crude fiber: 6–7%; Digestible energy: 3.5%; Crude protein: 19.16%) and low-fiber diets (Crude fiber: 2–3%; Digestible energy: 3.49%; Crude protein: 19.15%). Wheat bran fiber was purchased from Chengdu Tubeite Technology Co., Ltd. (Manufacturer: JRS, Model: WF200, purity >95%). All pigs were housed in one barn, each pig was housed in a separate pen, each pen was equipped with a feeder and a nipple drinker, room temperature: 28°C, free drinking and eating, the experimental period was 28 days. Pigs were slaughtered on the last day of the experiment and colonic contents were collected.

### DNA extraction, library construction, and metagenomic sequencing

Total genomic DNA was extracted from Colon content samples using the E.Z.N.A.® Soil DNA Kit (Omega Bio-tek, Norcross, GA, U.S.) according to manufacturer’s instructions. Concentration and purity of extracted DNA was determined with TBS-380 and NanoDrop2000, respectively. DNA extract quality was checked on 1% agarose gel.

DNA extract was fragmented to an average size of about 400 bp using Covaris M220 (Gene Company Limited, China) for paired-end library construction. Paired-end library was constructed using NEXTFLEX Rapid DNA-Seq (Bioo Scientific, Austin, TX, USA). Adapters containing the full complement of sequencing primer hybridization sites were ligated to the blunt-end of fragments. Paired-end sequencing was performed on Illumina NovaSeq/Hiseq Xten (Illumina Inc., San Diego, CA, USA) at Majorbio Bio-Pharm Technology Co., Ltd. (Shanghai, China) using NovaSeq Reagent Kits/HiSeq X Reagent Kits according to the manufacturer’s instructions.^1^ Sequence data associated with this project have been deposited in the NCBI Short Read Archive database (Accession Number: PRJNA849732).

### Sequence quality control and genome assembly

The data were analyzed on the free online platform of Majorbio Cloud Platform.^2^ Briefly, the paired-end Illumina reads were trimmed of adaptors, and low-quality reads (length < 50 bp or with a quality value <20 or having N bases) were removed by fastp (Chen et al., 2018).^3^

Metagenomics data were assembled using MEGAHIT (Li et al., 2016),^4^ which makes use of succinct de Bruijn graphs. Contigs with a length ≥ 300 bp were selected as the final assembling result, and then the contigs were used for further gene prediction and annotation.

### Gene prediction, taxonomy, and functional annotation

Open reading frames (ORFs) from each assembled contig were predicted using Prodigal/MetaGene.^5^ The predicted ORFs with a length ≥ 100 bp were retrieved and translated into amino acid sequences using the NCBI translation table.^6^

A non-redundant gene catalog was constructed using CD-HIT (Fu et al., 2012)^7^ with 90% sequence identity and 90% coverage. High-quality reads were aligned to the non-redundant gene catalogs to calculate gene abundance with 95% identity using SOAPaligner.^8^

Using Diamond software, the target amino acid sequences were compared with the PHI database by BLASTP,^9^ and the annotation results were obtained by combining the gene of the target species and its corresponding functional annotation information. If there is more than one result for each sequence, the best result is retained as the annotation result for that gene. The BLAST results are provided in M8 format. Finally, the gene abundance corresponding to each phenotypic classification was counted.

### Statistics

Data preprocessing was performed using Excel 2019 (Microsoft, USA), and data statistics were performed using SPSS 22.0 (IBM Corp, USA). Graphical display of results was performed using GraphPad Prism 8. RPKM value was used for heat map data, *Z-score* was used for data standardization, and average clustering was used for cluster analysis. Principal coordinate analysis (PCoA), principal component analysis (PCA) and NMDS analysis were performed using the normalized abundance values of the fungus communities with the vegan package in R software. The mulberry graph is drawn using the plotly package in the R software. Circos analysis was produced and analyzed using the tools of the Megi Bio cloud platform.

RPKM (Reads Per Kilobase Million):


$$RPKM_{i}=\frac{R_{i}*10^{6}}{L_{i}*\sum_{1}^{n}\left(R_{j}\right)}$$

$R_{i}$ represents the abundance value of Genei in a sample, that is, the number of reads aligned to Genei in the sample; $L_{i}$ represents the nucleotide length of Genei; $\overset{n}{\underset{1}{\sum }}\left(R_{j}\right)$ represents the sum of the reads corresponding to all genes in the sample.

Experimental Design Using a 3 × 2 design, Two-ANOVA was used to analyze two main effects and interaction effects. Results were expressed as mean ± standard error, and *p* < 0.05 indicated a significant difference.

## Results

### Effects of dietary fiber and genetics on fungal diversity in pig colon

**Figure 1:**
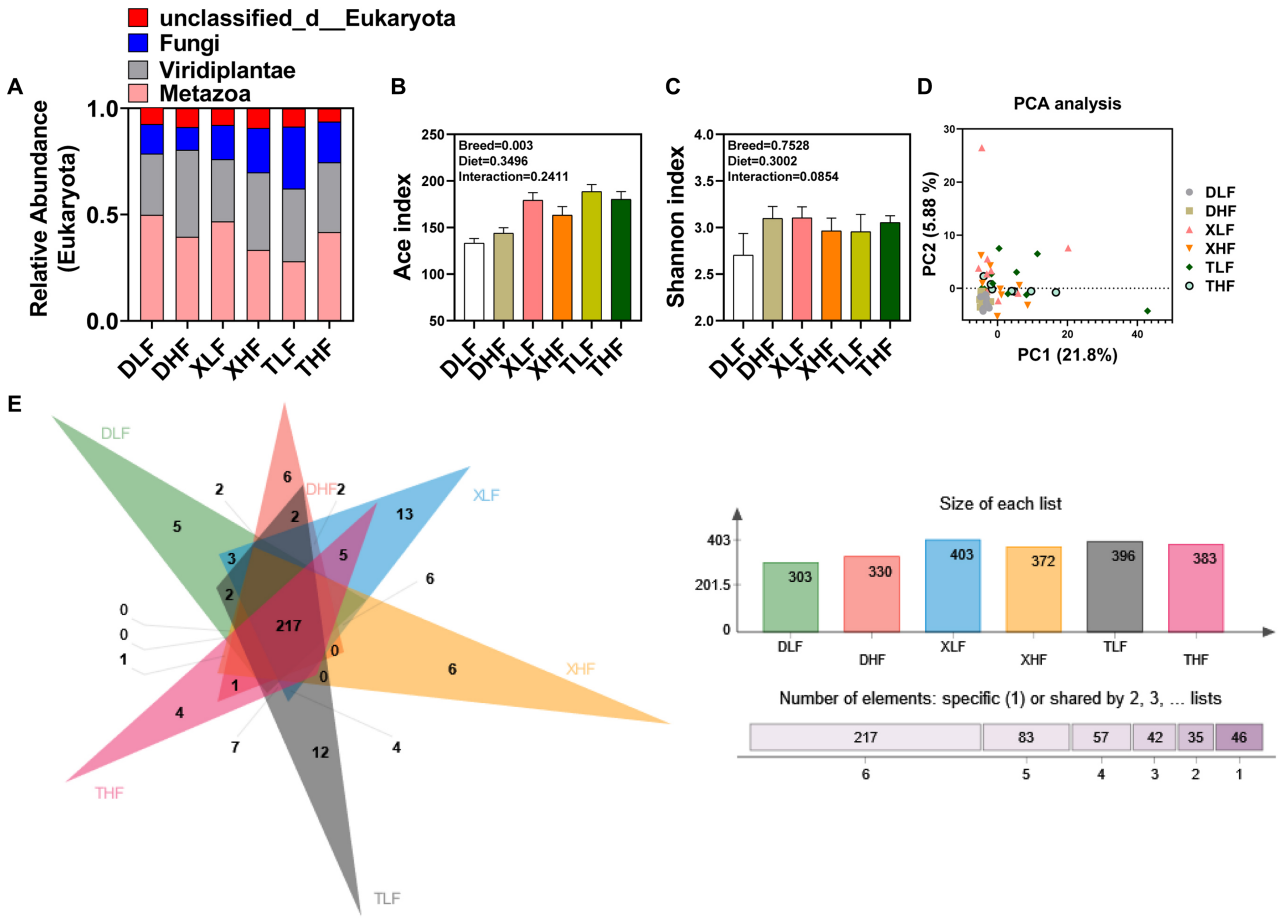
Diversity of fungi in the colon of Duroc, Xiangcun and Taoyuan pigs at different fiber levels. **(A)** Microbial composition in eukaryotes. **(B,C)** ACE and Shannon indices. **(D)** Unweighted UniFrac distance principal coordinate analysis (PCA) of fungi in different groups of pig colons. **(E)** Venn plot of species-level fungi in different groups.

**Figure 2:**
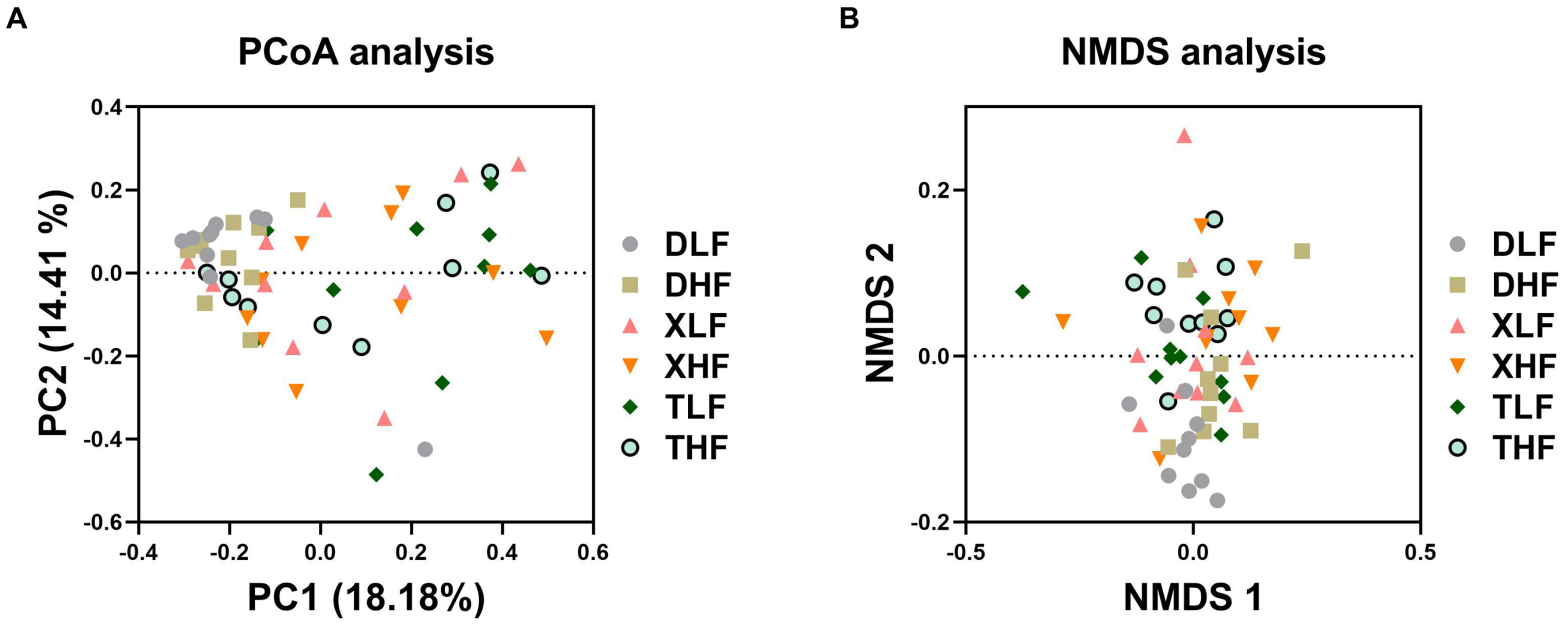
β-diversity of fungi in the colon of Duroc, Xiangcun and Taoyuan pigs at different dietary fiber levels. Principal coordinate analysis (PCoA) **(A)** and Nonmetric multidimensional scaling (NMDS) analysis **(B)**.

A total of 6,560,217 high-quality fungal-related sequences were detected in all samples, with an average length of 524.57 bp. The relative abundance of fungi in the colon of Taoyuan and Xiangcun Pigs was higher than that of Duroc pigs (Figure 1A). ACE and Shannon index were calculated to reflect the diversity of α in Colon. The Ace index of colonic fungi among different breeds was significantly different, and Taoyuan pigs were higher than Duroc pigs (*p* < 0.05) (Figure 1B). There was no significant effect of fiber diet and breed on the Shannon index (Figure 1C). Through PCA analysis, we found a high degree of sample overlap between the different groups (Figure 1D). To further explore the differences in the distribution of species-level fungi among the different groups, we performed Veen plot analysis and found that species-level fungi were the least numbers in Duroc’s colon and the most numbers in the XLF and TLF groups, there are 13 and 12 species, respectively (Figure 1E). PCoA plots based on the Bray Curtis (and unweighted UNIFRAC) distance matrix showed that the Taoyuan Pig and Xiangcun pig samples were highly overlapping. In contrast, the Duroc samples were relatively independent (Figure 2A). To overcome the shortcomings of the linear model (PCoA) and better reflect the nonlinear structure, we used NMDS stress values to evaluate the model’s accuracy. We found that NMDS results were similar to PCoA results (Figure 2B).

### Effects of fiber diet and genetics on the community and composition of porcine colonic fungi

**Figure 3:**
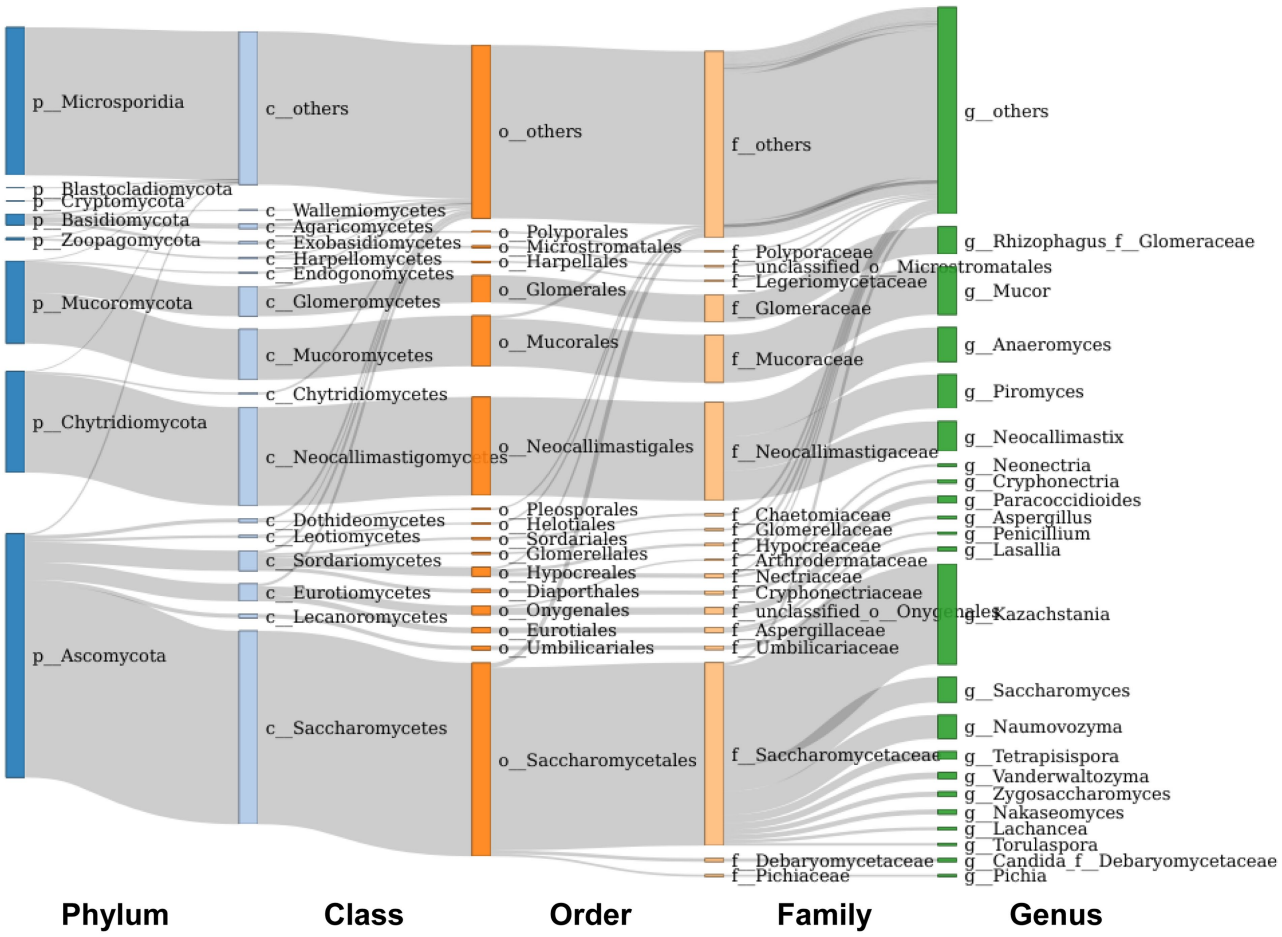
Composition of fungal microbes in the colon at the phylum-genus level.

**Figure 4:**
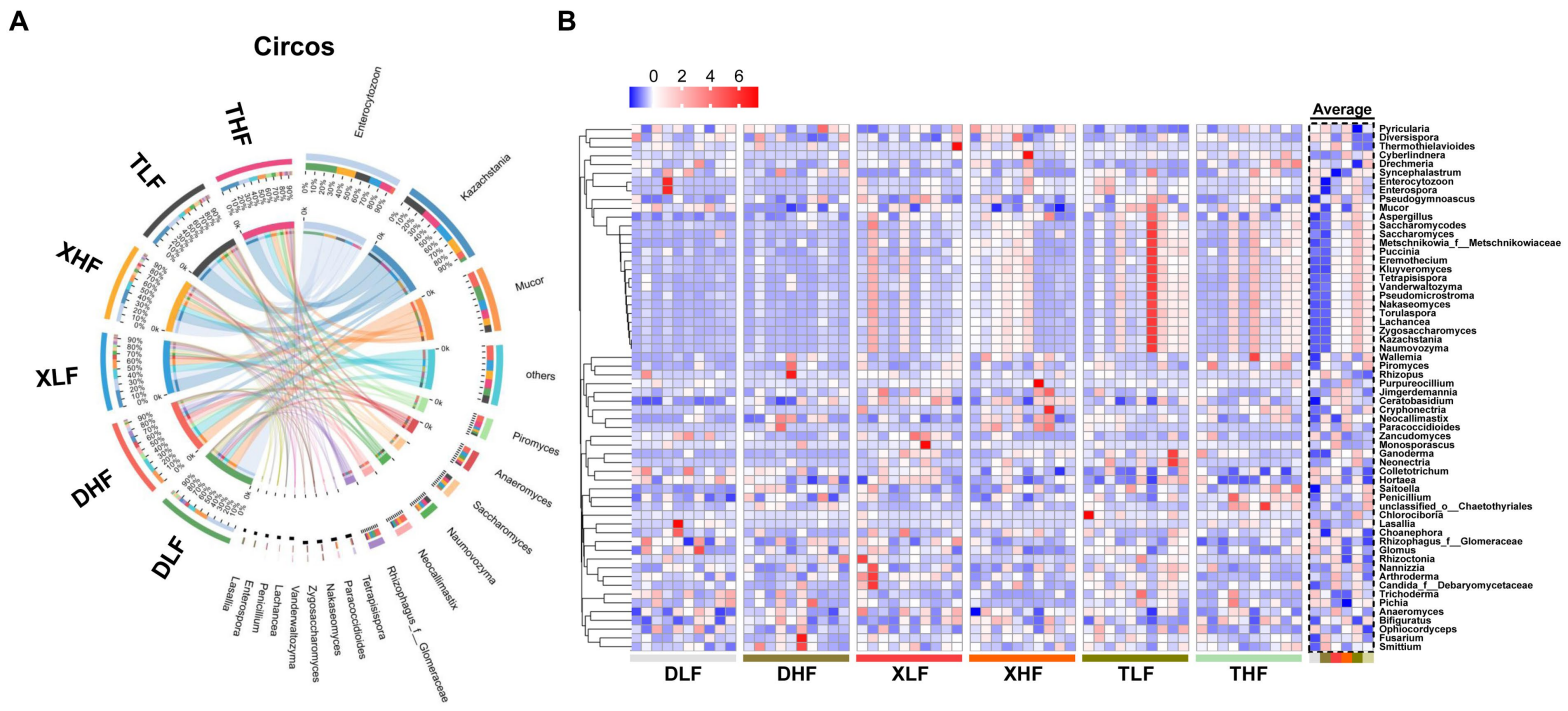
Fungal composition in the colon of Duroc, Xiangcun and Taoyuan pigs at different dietary fiber levels. **(A)** Circos plots of different samples and Genus at the phylum level. **(B)** Heat map of relative abundance of fungi at the species level for the top 40 abundances in colon contents of different samples and groups.

The main taxonomic composition of colonic fungi was investigated at the phylum and genus levels. A total of eight phylum-level fungi (*Ascomycota*, *Mucoromycota*, *Chytridiomycota*, *Zoopagomycota*, *Basidiomycota*, *Blastocladiomycota*, *Cryptomycota*, and *Microsporidia*) were identified in all samples (Figure 3). *Ascomycota*, *Mucoromycota*, *Microsporidia* and *Chytridiomycota* were identified as the main phylum-level fungi in the colon of different pig breeds, while *Ascomycota* was the most predominant phylum-level fungus in the colon of Taoyuan and Xiangcun pigs. The predominant phylum-level fungus in the pig colon was *Ascomycota*, but the *Microsporidia* phylum was the predominant phylum-level fungus in the low-fiber-fed Duroc pig colon (Supplementary Figure S1). Interestingly, the proportion of *Microsporidia* phylum in the colon of Duroc pigs was decreased when compared with a high-fiber diet compared to a low-fiber diet (Supplementary Figure S1). At the genus level fungi, we identified a total of 281 identifiable fungal genera in all samples. The main genus-level fungi were *Kazachstania*, *Mucor*, *Anaeromyces*, *Piromyces* and *Neocallimastix* (Figure 3). To further analyze the effect of fiber and species on fungal abundance at the genus level, we counted the fungal genera with the top 60 relative abundances. Among species, excluding undefined fungal genera, we found that 6 fungi at the genus level were significantly different (*Nakaseomyces*, *Zygosaccharomyces*, *Aspergillus*, *Ganoderma*, *Trichoderma*, and *Ceratobasidium*), and all showed an average intracolonal the highest relative abundance. A high-fiber diet significantly decreased the relative abundances of *Mucor*, *Colletotrichum*, *Hortaea* and *Arthroderma* and significantly increased the relative abundances of *Neocallimastix*, *Paracoccidioides*, *Pyricularia*, *Wallemia* and *Neonectria* in pig colon (Figures 4A,B; Supplementary Table S1).

### Host pathogenicity-associated genes carried by fungi in the colon

**Figure 5:**
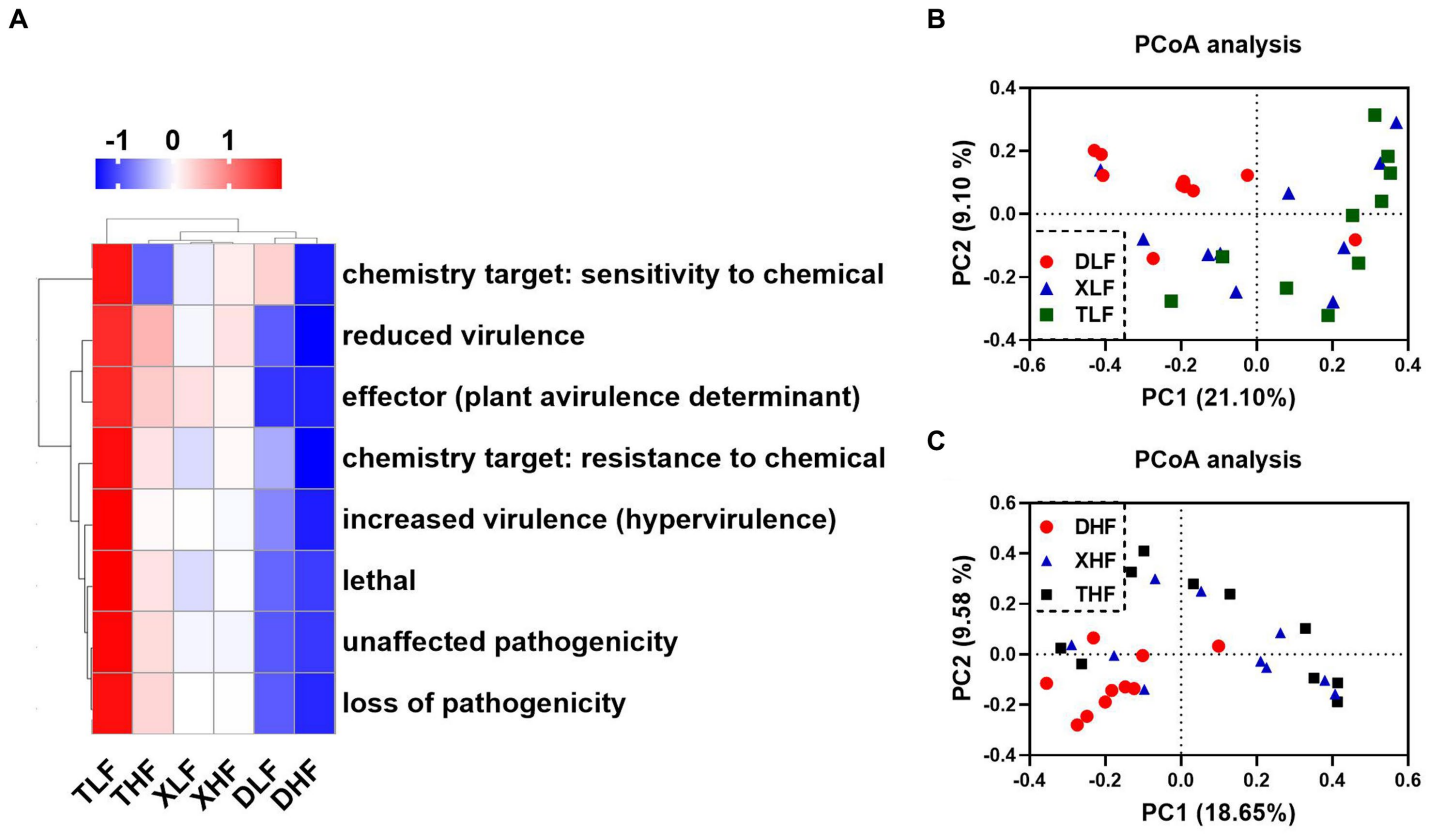
Differences in host-associated virulence factors carried by fungal profiles in the colon of Duroc, Xiangcun and Taoyuan pigs at different dietary fiber levels. Heat map of relative abundance of PHI Phenotype in colonic contents of different samples and groups **(A)**. PCoA analysis plots of host-associated virulence factors carried by fungi at low **(B)** and high **(C)** fiber levels in different breeds.

**Figure 6:**
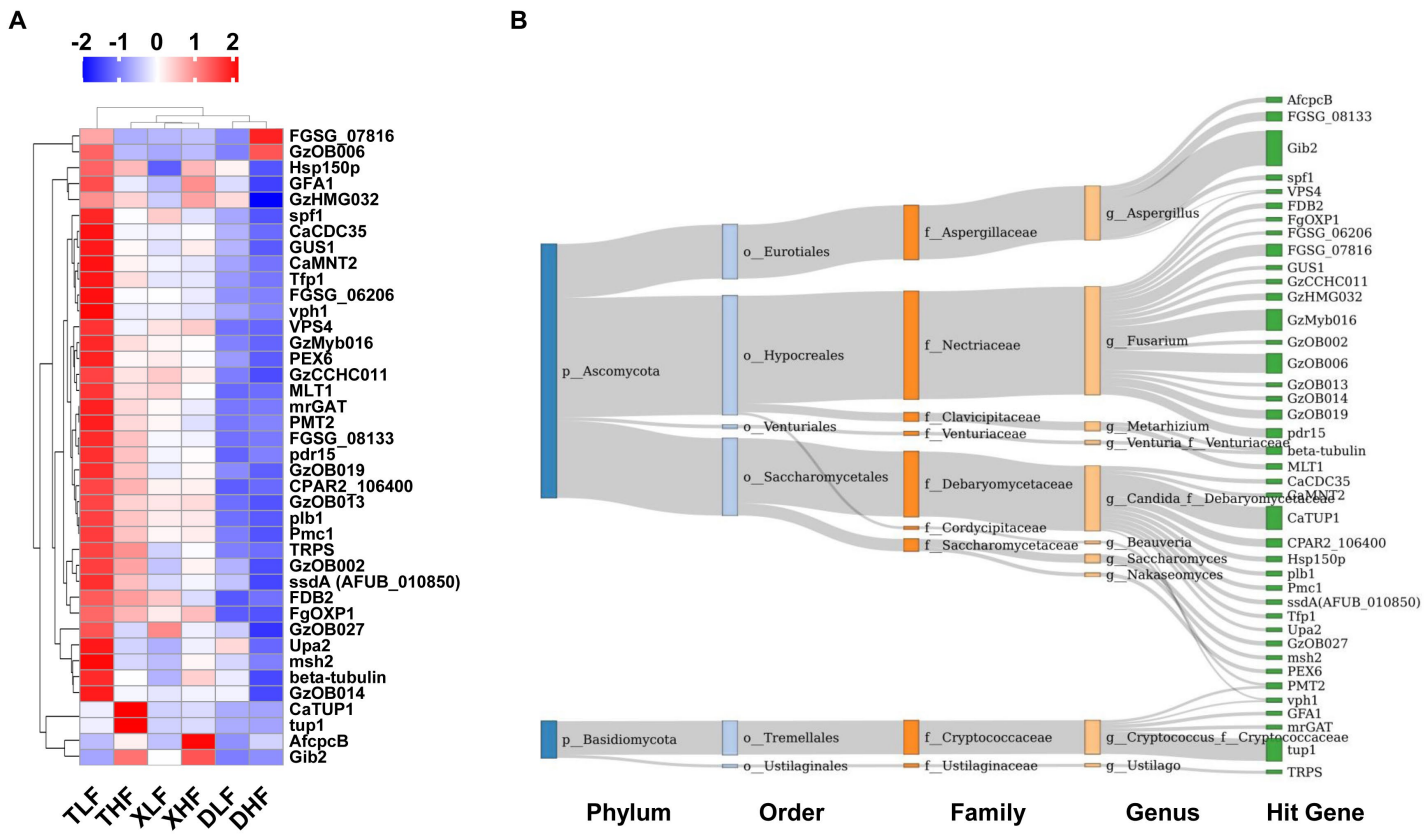
Composition of host-associated pathogenic genes carried by fungi in the colonic contents of Duroc, Xiangcun and Taoyuan pigs fed different fiber levels. **(A)** Heat map of relative abundance of Top 40 pathogenicity-associated genes. **(B)** The distribution of Top 40 pathogenic genes on fungal microbes at different taxonomy levels. The colors of the rectangles represent different taxonomy levels. The length of the rectangles indicates the number of pathogenic genes.

The proteins encoded by genes carried by fungi associated with host pathogenicity are important for pathogenic fungi in infecting hosts and escaping host immunity. The Pathogen-Host Interaction Database (PHI-base) catalogs experimentally validated pathogenicity, virulence and effect genes of fungal, oomycete and bacterial pathogens from animal, plant, fungal and insect hosts. We took the target amino acid sequences by BLASTP, compared them with the PHI database, and combined the genes of the target species with their corresponding functional annotation information to obtain the annotation results. The main categories of genes associated with host pathogenicity in porcine colon were reduced virulence, unaffected pathogenicity, loss of pathogenicity and lethal. The highest abundance was found in the colon of low-fiber Taoyuan pigs, while the lowest abundance was found in the colon of Duroc pigs (Supplementary Table S2). The results of the heat map showed that the high fiber diet reduced the abundance of different categories of host pathogenicity-related genes in the intestine of Taoyuan pigs (Figure 5A). Based on PCoA analysis, we found that samples from Taoyuan and Xiangcun pigs were highly overlapping, while samples from Duroc pigs were more independent, which was similar to the PCoA results for fungi in the colon (Figures 5B,C). We further analyzed the top 40 abundance of host pathogenicity-related genes carried by the fungus, and we found that *CaTUP1*, *tup1*, *GzMyb016*, *pdr15*, *FGSG_08133*, *GzOB019*, *CPAR2_106400*, *FDB2*, *spf1*, *ssdA (AFUB_010850)*, *CaCDC35*, *plb1*, *GzCCHC011*, *Tfp1*, *GzOB013*, *CaMNT2*, *FGSG_06206* and *FgOXP1* all had significant between-species differences. *GzOB027* had significant differences between different levels of fiber diet (Figure 6A; Supplementary Table S3). By analyzing the origin of the host pathogenicity-related genes in the top 40 abundance, it was found that the main phylum-level microorganisms carrying host pathogenicity-related genes in the pig colon were *P_Ascomycota* and *P_Basidiomycota*, while *g_Aspergillus*, *g_Fusarium*, *g_Candida_f_Debaryomycetaceae*, *g_Cryptococcus_f_Cryptococcaceae*, *g_Metarhizium*, and *g_Saccharomyces* are the major genus-level microorganisms (Figure 6B).

## Discussion

As the “microbial organ” of mammals, intestinal microorganisms mediate nutrient absorption and metabolism, development of the intestinal immune system, and maintenance of the intestinal mucosal barrier (de Vos et al., 2022). In addition to commensal bacteria, fungi are increasingly acknowledged as significant contributors to mammalian intestinal health, nutrient absorption, and their potential for pathogenicity (Kapitan et al., 2019). In this study, we investigated the distribution of fungi in the intestine of different pig breeds and crosses, namely commercial breed (Duroc pigs), Chinese local breed (Taoyuan pigs), and a hybrid of the two (Xiangcun pigs), as well as the impact of dietary fiber on fungal composition. Moreover, we analyzed the host-associated pathogenicity genes carried by the fungi using PHI-base and evaluated the influence of genetics and fiber diet on these genes.

The analysis of our results based on community and different species revealed marked differences in fungal composition at the phylum and genus level in the intestine of Taoyuan and Duroc pigs, indicating that genetic background exerts a significant influence on fungal diversity in the gut. Furthermore, our findings showed that Xiangcun pigs, as a hybrid, exhibited a mixed fungal profile, displaying characteristics of both Duroc and Taoyuan pigs, suggesting that intestinal fungi can be vertically transmitted *via* heterozygous inheritance, as previously reported (Moeller et al., 2018). Surprisingly, we did not observe any significant impact of dietary fiber on the abundance and diversity of intestinal fungi. Contrary to our findings, several researchers have discovered that feed fermented by *lactobacillus*, specifically bran feed, markedly diminishes the fungal diversity in growing pig manure. This reduction also impacts the abundance of certain fungal genera, including *Wallemia*, *Trichosporon*, *Candida*, and *Aspergillus* (Zhang et al., 2023). This discrepancy might be attributed to the fact that variations in the specific type and source of fiber diets can lead to differential effects on fungal communities. Since different types of fiber might offer distinct nutritional values or create varying growth conditions for fungi, their influence on fungal populations can differ.

At the phylum level, the dominant fungal taxa found in the intestines of various pig breeds were *Ascomycota*, *Chytridiomycota*, *Mucoromycota*, and *Microsporidia*. These findings differ somewhat from the most prevalent fungal phyla reported in previous studies of pig, mouse, and human intestines, likely influenced by variations in pig age and advancements in technology (Scupham et al., 2006; Hoffmann et al., 2013; Li et al., 2020). Notably, the abundance of *Ascomycota* in the intestine of Taoyuan and Xiangcun pigs was higher than that of Duroc pigs, Ascomycota are important decomposers in the ecosystem, breaking down macromolecules such as cellulose or lignin, and the high abundance of *Ascomycota* in the intestine of Taoyuan and Xiangcun pigs may be related to their trait of roughage tolerance (Challacombe et al., 2019). At the genus level, *Kazachstania*, *Enterocytozoon*, *Mucor* and *Saccharomyces* were the most dominant genera. One study found that *Loreleia*, *Russula*, *Nephroma* and *Candida* were the predominant genus-level fungi in the feces of different species of pigs after examining the structure of their feces, which may be due to regional differences in gut microbial populations due to the functional heterogeneity of feces and different gastrointestinal tract (GIT) segments, and also due to the low biodiversity and abundance of gut fungal communities, which are more influenced by environment, species and sequencing techniques (Arfken et al., 2019; Martinez-Guryn et al., 2019; Li et al., 2020). Moreover, there is a trend toward breed-specific differences in fungal genera, with Taoyuan pigs exhibiting the highest mean abundance of *Kazachstania*, *Mucor*, and *Saccharomyces*, while a high-fiber diet reduced the abundance of *Mucor*. *Kazachstania* has been reported to be the predominant fungal genus in the intestinal tract of weaned piglets, which is consistent with our findings, where it has been shown that *Kazachstania* can play a crucial role in intestinal glycolytic metabolism through lysine desuccinylation (Arfken et al., 2019; Summers et al., 2019; Hu et al., 2023). *Saccharomyces* has been found to enhance piglet immunity and reduce oxidative stress, which is crucial for piglet health (Sauerwein et al., 2007; Liu et al., 2017), and interestingly, related studies also demonstrated that the levels of IgG and IgM associated with intestinal mucosal homeostasis were significantly higher in the plasma of Taoyuan pigs than in Duroc pigs, so *Saccharomyces* in the intestine of Taoyuan pigs may be improving the intestinal homeostasis of local native pig breeds by improving their disease resistance (Ding et al., 2023). On the other hand, limited studies have linked *Mucor* with mucormycosis and pneumonia in pigs (Evans et al., 2018). The high proportion of *Mucor* in the intestine of Taoyuan pigs might be related to their genetic background. Conversely, dietary fiber promotes intestinal motility and reduces the colonization of harmful bacteria (Capuano, 2017), which could explain the observed reduction in *Mucor* abundance with high-fiber diets.

Infectious diseases are a major threat to the health of plants, animals, humans and entire ecosystems (Brown et al., 2012). Local and global infectious diseases pose significant risks to food cultivation, feed security, and livestock farming (Fisher et al., 2018). The Pathogen-Host Interaction Database (PHI-base) manually curates experimentally validated pathogenicity, virulence, and effector genes from fungal, bacterial, and protozoan pathogens that infect animal, plant, fish, insect, and/or fungal hosts (Urban et al., 2022). PHI-base aims to provide detailed phenotypic information on pathogenicity and effector genes and their interactions with hosts. This is important for exploring the pathogenicity of pathogenic bacteria in animal production, as well as for designing antiviral drugs or live attenuated vaccines. For instance, the African swine fever virus has been discovered to evade host antiviral innate immune responses, including the modulation of inflammatory reactions and interferon production, through MGF360. Notably, when the expression of MGF360 in the African swine fever virus is suppressed, its pathogenicity is significantly diminished (Li et al., 2021).

In this study, we investigated diet- and genetic-mediated changes in pathogenicity-related genes carried by fungi in the pig colon by comparing fungal genes in pig colon with the PHI-base database. Our results revealed that the abundance of PHI phenotypes was highest in the colon of Taoyuan pigs, a local Chinese pig breed. A high-fiber diet reduced the abundance of PHI phenotypes in the colon of Taoyuan pigs, but did not affect the abundance of PHI phenotypes in the colon of Duroc pigs and Xiangcun pigs, a crossbred pig. We speculate that this may be due to the long-term field rearing of the parents of Taoyuan pigs, which were often exposed to a variety of complex fungal environments, including wild plants, resulting in a more abundant pathogenicity-related gene in the intestine of Taoyuan pigs. Additionally, we found that genes associated with plant pathogenicity [*tup1* (Elías-Villalobos et al., 2011), *GzMyb016* (Son et al., 2011), *FGSG_08133* (Wang et al., 2011), *GzOB019* (Son et al., 2011), *FDB2* (Kettle et al., 2015), spf1 (Nguyen et al., 2008), *GzCCHC011* (Son et al., 2011), *GzOB013* (Son et al., 2011), *FGSG_06206* (Wang et al., 2011), *FgOXP1* (Yang et al., 2018)] were enriched in the colon of Taoyuan pigs, which was laterally verified. Although the large number of plant pathogenic genes carried by the fungus does not have a significant impact on pigs, with the accompanying fecal excretion, the plant-associated pathogenic genes of the fungus can also be a threat to the plants surrounding the pig breeding environment. *CaTUP1* (Murad et al., 2001), *CPAR2_106400* (Papp et al., 2018), *CaCDC35* (Rocha et al., 2001), *Tfp1* (Jia et al., 2014), and *CaMNT2* (Munro et al., 2005) have all been shown to be associated with the pathogenic process of candidiasis, and *ssdA (AFUB_010850)* (Thammahong et al., 2019) and *plb1* (Noverr et al., 2003) have been found to be associated with Pulmonary aspergillosis and fungal meningitis, and the reduced abundance of host disease-causing-related genes carried by these fungi in the intestine of crossbred Xiangcun pigs also suggests that we can reduce the abundance of harmful genes by genetic means, thus reducing the potential harm of host disease-causing-related genes carried by food-borne animals.

## Conclusion

Overall, our study explored the fungal profile in the intestine of different breeds of pigs under different dietary fiber diets and resolved potential dietary and genetic influences on fungal composition, function and host pathogenicity-related genes carried by the fungi. Our results showed that dietary fiber diet caused large changes in fungal structure in the colon of commercial Duroc pigs, but had no significant effect on intestinal fungal composition of rough-fed Taoyuan pigs. The fungal composition in the colon of commercial Duroc pigs and Taoyuan pigs, a local Chinese pig breed, had a large difference, while the fungal composition and function in the intestine of Xiangcun pigs, a cross between Taoyuan and Duroc pigs, tended to be mixed with that of the parents. This provides a partial reference for our later pig breeding. We also resolved the host pathogenicity-related genes carried by the fungal spectrum in the pig intestine for the first time, and found that the intestine of Taoyuan pigs, a local Chinese pig breed, was enriched with fungal pathogenicity-related genes, while the abundance of pathogenicity-related genes carried by the fungi in the intestine of Xiangcun pigs was reduced by crossing the offspring, our results are only a preliminary exploration of the host pathogenicity-related genes carried by the fungi in the pig intestine, but this also has the potential to provide researchers in The results are only a preliminary exploration of the host pathogenicity-related genes carried by fungi in the intestine of pigs, but it is also a reference for researchers in the development of antifungal drugs and to explore the reduction of host pathogenicity gene abundance carried by fungi in the intestine of food-borne animals.

## Data availability statement

The datasets presented in this study can be found in online repositories. The names of the repository/repositories and accession number(s) can be found in the article/Supplementary material.

## Ethics statement

The animal study was approved by Institutional Animal Care and Use Committee of Sichuan Agricultural University, Sichuan, China (No. 20181105). The study was conducted in accordance with the local legislation and institutional requirements.

## Author contributions

JH and XK conceived, designed and directed the experiment development, and the article’s writing. TW and JLi conducted animal experiments, visualized the results, and wrote manuscripts. YL, BY, PZ, ZH, XM, JY, JLu, and HY put forward constructive opinions on the results and discussion of the manuscript. All authors have read and agreed with the final manuscript.

## Footnotes

^1^ www.illumina.com

^2^ www.majorbio.com

^3^https://github.com/OpenGene/fastp, version 0.20.0.

^4^https://github.com/voutcn/megahit, version 1.1.2.

^5^ http://metagene.cb.k.u-tokyo.ac.jp/

^6^ http://www.ncbi.nlm.nih.gov/Taxonomy/taxonomyhome.html/index.cgi?chapter=tgencodes#SG1

^7^http://www.bioinformatics.org/cd-hit/, version 4.6.1.

^8^http://soap.genomics.org.cn/, version 2.21.

^9^http://blast.ncbi.nlm.nih.gov/Blast.cgi, Version 2.2.31+.
