# Corrigendum: Combined effects of host genetics and diet on porcine intestinal fungi and their pathogenic genes

**DOI:** 10.3389/fmicb.2023.1319760

**Published:** 2023-11-13

**Authors:** Tao Wang, Jiahao Liu, Yuheng Luo, Bing Yu, Xiangfeng Kong, Ping Zheng, Zhiqing Huang, Xiangbing Mao, Jie Yu, Junqiu Luo, Hui Yan, Jun He

**Affiliations:** ^1^Institute of Animal Nutrition, Sichuan Agricultural University, Chengdu, China; ^2^Key Laboratory of Animal Disease-resistant Nutrition, Chengdu, China; ^3^Institute of Subtropical Agriculture, Chinese Academy of Sciences, Changsha, China

**Keywords:** genetics, fungi, metagenomic, diet fiber, pigs

In the published article, there was an error in the Funding statement in that the funding information was not added to the manuscript. The correct Funding statement appears below.

## Funding

This study was supported by the National Natural Science Foundation of China (No. U20A2056) and the Key Research and Development Program of Sichuan Province (2020YFN0147).

The authors apologize for this error and state that this does not change the scientific conclusions of the article in any way. The original article has been updated.

