# Supplementary material for: Combined effects of host genetics and diet on porcine intestinal fungi and their pathogenic genes
Source: Front Microbiol. 2023 Sep 25;14:1192288. doi: 10.3389/fmicb.2023.1192288 (PMC10563851; doi:10.3389/fmicb.2023.1192288)
Supplement: Supplementary file 2 [file Data_Sheet_1.docx]

**Combined effects of host genetics and diet on porcine intestinal fungi and their pathogenic genes**

Tao Wang^1,2†^, Jiahao Liu^1,2†^, Yuheng Luo^1,2^, Bing Yu^1,2^, Xiangfeng Kong^3*^, Ping Zheng^1,2^, Zhiqing Huang^1,2^, Xiangbing Mao^1,2^, Jie Yu^1,2^, Junqiu Luo^1,2^, Hui Yan^1,2^, Jun He^1,2*^

^1^Institute of Animal Nutrition, Sichuan Agricultural University, Chengdu, Sichuan 611130, P. R. China

^2^Key Laboratory of Animal Disease-resistant Nutrition, Chengdu, Sichuan 611130, P. R. China

^3^Institute of Subtropical Agriculture, Chinese Academy of Sciences, Changsha, Hunan 611130, P. R. China 410125, P. R. China

^*^Corresponding author: Institute of Animal Nutrition, Sichuan Agricultural University, Chengdu, Sichuan 611130, P. R. China; Tel: +86-13419354223, Fax: +86-28-86291781, Email: hejun8067@163; comnnkxf@isa.ac.cn.

^†^ Contribute equally to this work.


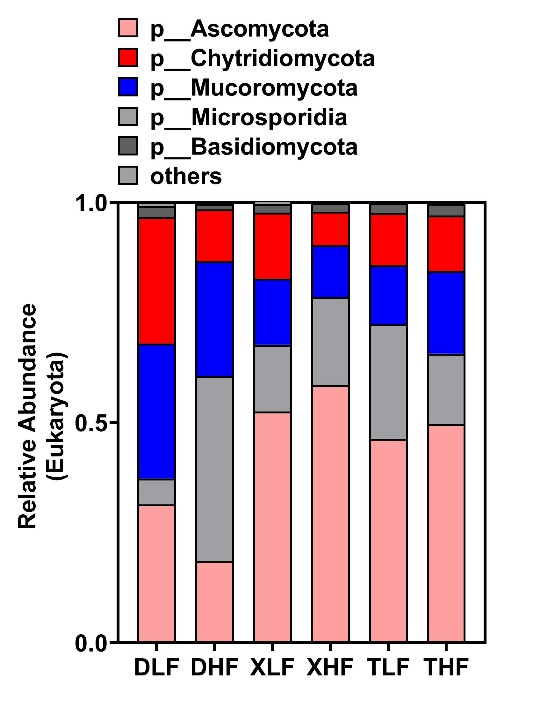


**Figure S1:** Effects of different dietary fiber diets and breeds on the composition of fungi at intestinal hilum level in pigs.
